# Continuous Remote Monitoring in Moderate and Severe COVID-19 Patients

**DOI:** 10.7759/cureus.44528

**Published:** 2023-09-01

**Authors:** Avinash H Rajanna, Vaibhav S Bellary, Sohani Kashi Puranic, Nayana C., Jatin Raaghava Nagaraj, Eshanye D A., Preethi K.

**Affiliations:** 1 General Medicine, Employees’ State Insurance Corporation and Medical College (ESIC-MC) and Post Graduate Institute of Medical Science and Research (PGIMSR) Model Hospital, Rajajinagar, Bangalore, IND

**Keywords:** early warning score, clinical deterioration, continuous monitoring, remote monitoring, covid-19, contactless

## Abstract

Background

COVID-19 steadily built up the pressure on healthcare systems worldwide, creating the need for novel methods to alleviate the burden. Continuous remote monitoring of vital parameters reduces morbidity and mortality in hospitals by providing real-time disease data that can be analyzed through web portals. It enables healthcare workers to identify which patients require prompt administration of healthcare. Patients remain under the purview of their doctors and can be notified early if there are any deteriorations in the parameters being monitored.

Aims

To evaluate the use of remote monitoring in moderate and severe COVID-19 patients and to correlate the Dozee Early Warning Score (DEWS) with severity and outcome in moderate and severe COVID-19 patients.

Materials and methods

We conducted a prospective study on adult (>18 years old) moderate and severe COVID-19 patients during the second wave of COVID-19. The vitals of the subjects were continuously monitored using Dozee, a contactless remote patient monitoring system enabled with DEWS that reflects the overall patient condition based on respiratory rate (RR), heart rate (HR), and oxygen saturation (SpO_2_). We assessed the correlation of DEWS with patients’ clinical outcomes: deteriorated or recovered.

Results

Thirty-nine COVID-19 patients were recruited for the study, of whom 29 were discharged after recovery and 10 deteriorated and died. Respiratory rate trend, respiratory rate DEWS, SpO_2_ DEWS, and total DEWS showed a significant reduction in recovered patients, while the same parameters showed a significant increase followed by consistently high scores in patients who deteriorated and died due to the disease. Total DEWS was proportional to the risk of mortality in a patient.

Conclusion

We concluded that continuous vitals monitoring and the resulting DEWS in moderate and severe COVID-19 patients were indicators of their improvement or deterioration. DEWS uses continuous remote monitoring of routinely collected vitals (HR, RR, and SpO_2_) to serve as a predictor of patient outcome.

## Introduction

The COVID-19 pandemic has had a tremendous impact on the world, having devastated an alarming number of lives. It exposed the gaps in the traditional healthcare delivery systems: with healthcare professionals exposed to the disease from patients, not only are they at a high risk of nosocomial transmission, but they are also overburdened [1,2]. The disease has thus overwhelmed the existing healthcare infrastructures of developing and developed countries alike [3,4]. This has resulted in a delay in diagnosing the deterioration of patients, which is a determinant of increased mortality [5].

In the context of this pandemic, health monitoring is gaining significant traction and has emerged as a key area of interest in recent times [6]. Continuous monitoring of vital parameters reduces morbidity and mortality in hospitals, while remote monitoring is capable of reducing the potential spread and burden on healthcare systems [4,6]. Contactless sensors are perfect amalgamations of the two - physicians can access relevant data from patients at different time points, allowing for continuous monitoring of patients while eliminating the need for proximity [7]. Contactless measurement of vitals can be achieved by incorporating the principle of ballistocardiography (BCG). BCG captures mechanical vibrations produced by body movements that translate into the recording of vital parameters like heart rate (HR), respiratory rate (RR), oxygen saturation (SpO_2_), etc. [8,9].

Previously, in order to prioritize and allocate resources in intensive care units (ICUs) [10], the concept of Early Warning Score (EWS) was developed. EWSs take into consideration a patient’s vital parameters and their deviation from normal limits. A higher score indicates a higher mortality rate, and the score itself determines the type of warning triggered and the suitable response to be initiated [11].

COVID-19 provoked the need for a newer, easier, and logistically viable monitoring system at even a grassroots level, in contrast to the traditional in-person model [12]. Remote monitoring of vital parameters in COVID-19 patients with non-serious symptoms can help reduce the burden on healthcare facilities and make them available for high-risk groups and the seriously affected [4]. Dozee is a device that employs the principle of BCG to monitor vital parameters. It digitalizes patients’ vitals, which can be accessed remotely by the physician, who can assess the trends of these parameters over time. With its own EWS, the Dozee Early Warning Score (DEWS) can act as a harbinger of patient deterioration [13]. This study aims to assess the utility of the DEWS for predicting clinical deterioration and its correlation with the severity and outcome of COVID-19 patients.

## Materials and methods

Ethical clearance for the study was obtained from the Institutional Ethics Committee. The data were collected for a period of one year, from August 2020 to July 2021.

Inclusion and exclusion criteria

Inclusion Criteria

Adult patients (>18 years) with either reverse transcriptase polymerase chain reaction (RT-PCR) or rapid antigen test (RAT) positive for COVID-19 in moderate and severe categories were included.

Moderate COVID-19 patients were defined as those with SpO_2_ of 90-94% at room air or RR of 24-30 cycles per minute (cpm), while severe COVID-19 patients were defined as those with SpO_2_ <90% at room air or RR ≥ 30 cpm [14].

Exclusion Criteria

Mild COVID-19 cases, defined as patients with SpO_2_ >94% or RR < 24 cpm [14], were excluded.

**Figure 1:**
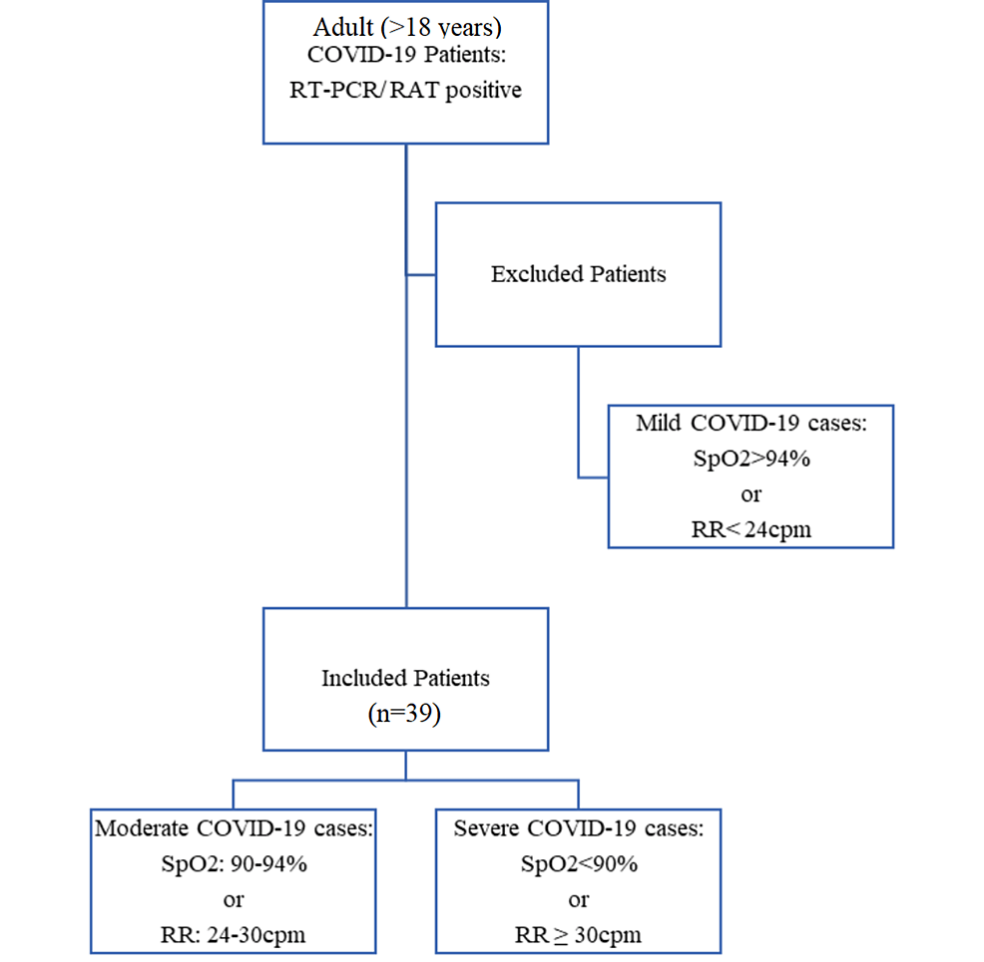
Inclusion and exclusion criteria for the study. RT-PCR: reverse transcriptase polymerase chain reaction, RAT: rapid antigen test, SpO_2_: oxygen saturation, RR: respiratory rate, cpm: cycles per minute, n: number of patients.

Based on these criteria (Figure 1), 39 patients were recruited for the study.

Variables monitored

Dozee

Dozee, a contactless remote patient monitoring system, is a device that uses ballistocardiography to sense patients' heartbeats, breaths, and body movements. It is installed under the mattress of the bed, which then monitors micro-vibrations produced by the body during sleep. The device employs proprietary algorithms to convert muscular, respiratory, and cardiac movements into recordable vital parameters (RR and HR). It comes with the added accessory of a pulse oximeter that is used to measure SpO_2_ [13].

The vitals of the subjects were continuously monitored by Dozee, and these datasets were collected. The ranges for heart rate and respiratory rate are 30-170 ± 3 beats per minute (bpm) and 6-45 ± 2 cycles per minute (cpm), respectively. The movement and bed-exit accuracy are both over 80% [15]. Each vital parameter is scored on a scale of 0-3, as shown in Table 1.

**Table 1:** Scoring of vital parameters by Dozee. HR: heart rate, RR: respiratory rate, SpO_2_: oxygen saturation.

Vital Parameter	0	1	2	3
HR	51–90 bpm	41–50 and 91–110 bpm	111–130 bpm	≤40 and ≥130 bpm
RR	12–20 cpm	9–11 cpm	21–24 cpm	≤8 and ≥25 cpm
SpO_2_	≥96%	94–95%	92–93%	≤91%

Dozee Early Warning Score

Dozee is equipped with an early warning system, the DEWS, which alerts healthcare workers if a patient's vitals are deteriorating, reflecting the overall patient condition. DEWS is a cumulative score of HR, RR, and SpO_2_ scores.

Individual scores are added for an overall DEWS total, ranging from 0 to 15. A score of 4 or more triggers an alert for immediate review and intervention. A total DEWS score of 2-3 or an individual score of 3 for any vital parameter indicates moderate risk and requires an urgent ward-based response, while a score of 0-1 indicates low risk and the response to this alert can be ward-based [13].

The data from the continuous monitoring of the RR, HR, and SpO_2_ of the 39 patients were analyzed for their duration of stay at the hospital. The DEWS of patients was also analyzed. There are no conflicts of interest.

Patient outcomes

The patients were monitored, and the outcome was measured either as recovered (clinical improvement, decreasing trend of DEWS score, and discharge) or deteriorated (clinically worsening, increasing trend of DEWS score, and death of the patient).

The patients were discharged according to the Government of Karnataka discharge protocol after 10 days of symptom onset when the following criteria were met: no fever/symptoms for the last three consecutive days before discharge (without antipyretics), SpO_2_ above 95% for the last four consecutive days (without oxygen support), resolution of clinical signs/symptoms (based on the report of investigations), resolution of breathlessness, repeat inflammatory markers (serum ferritin, lactate dehydrogenase, D-dimer, C-reactive peptide) in normal ranges/decreasing trends at the time of discharge, one negative RT-PCR/cartridge-based nucleic acid amplification test (CBNAAT)/Truenat done after three days following complete clinical recovery. The patients were discharged upon meeting these criteria. If the test report was positive, the swab test was repeated after 72 hours [16,17].

## Results

Age distribution

**Figure 2:**
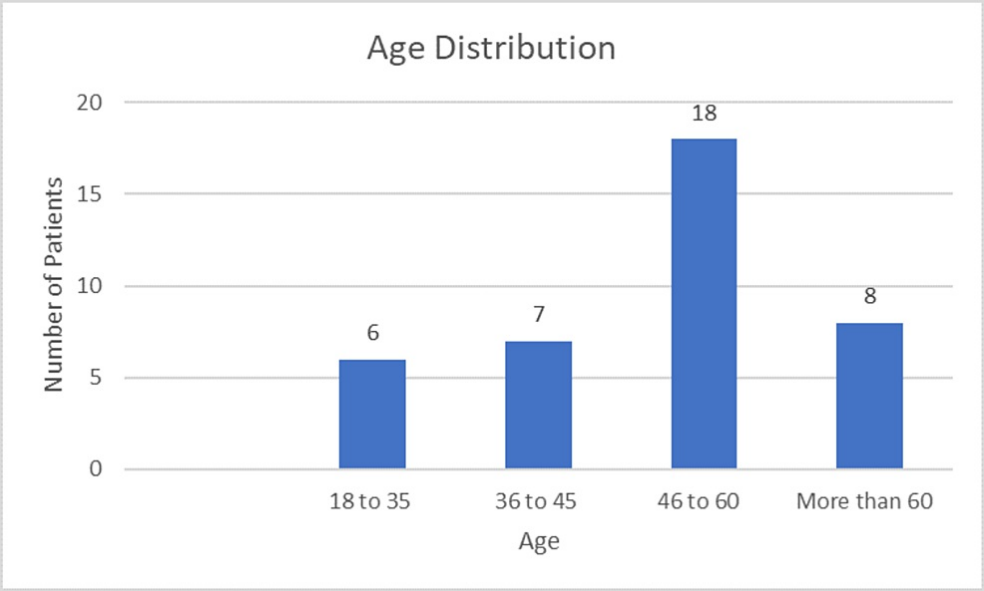
Age distribution of the patients.

Figure 2 shows that out of the 39 patients recruited for the study, six were in the age group of 18-35 (15%), seven in 36-45 (18%), 18 in 46-60 (46%), and eight patients were above 60 years of age (21%).

Gender distribution

**Figure 3:**
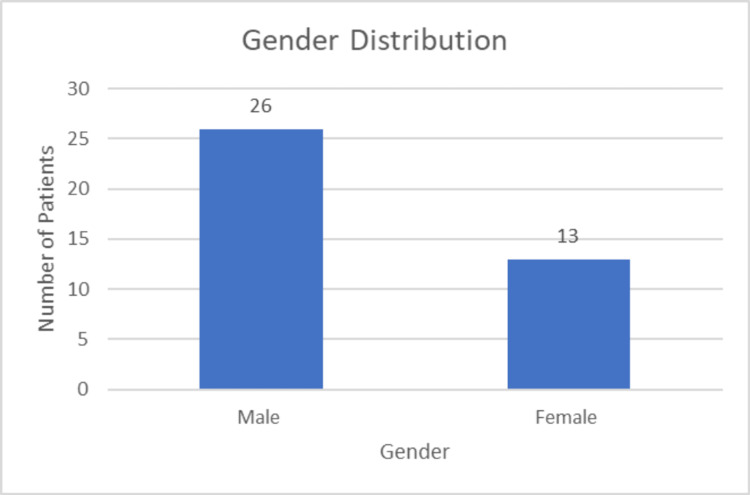
Distribution of gender.

Figure 3 shows that out of the 39 patients, 26 were male (66.67%) and 13 were female (33.33%).

Comorbidities

**Figure 4:**
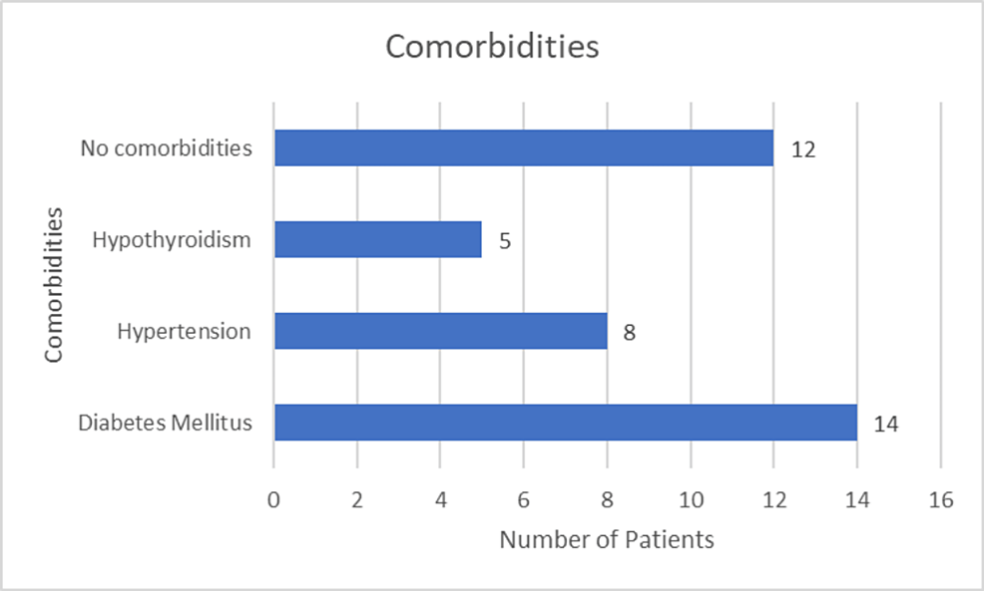
Distribution of comorbidities.

Figure 4 shows that out of the 39 patients, 12 had no comorbidities (30.8%), while 27 had various comorbidities (69.2%). Five patients had hypothyroidism, eight had hypertension, and 14 had diabetes mellitus.

DEWS graphs: recovered

The following graphs show the various DEWS and vitals trends in a patient who recovered and was discharged from the hospital. The average hospital stay for the patients was five days.

*Heart Rate DEWS*

**Figure 5:**
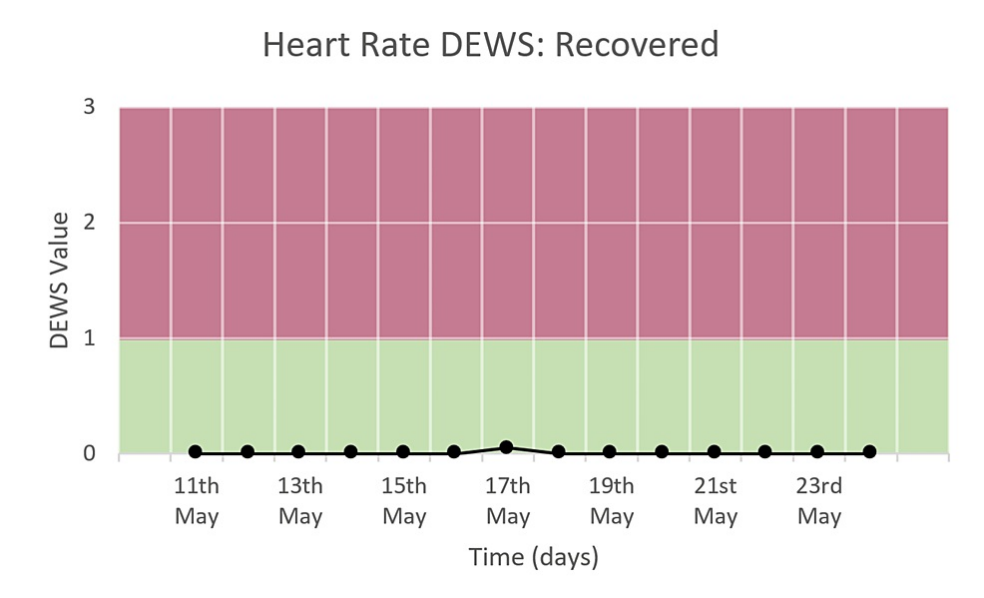
Heart rate DEWS of a patient who recovered and was discharged.

The heart rate DEWS is shown in Figure 5. There was no change in the trend during the study period. The DEWS score is consistently 0.

Respiratory Rate DEWS

**Figure 6:**
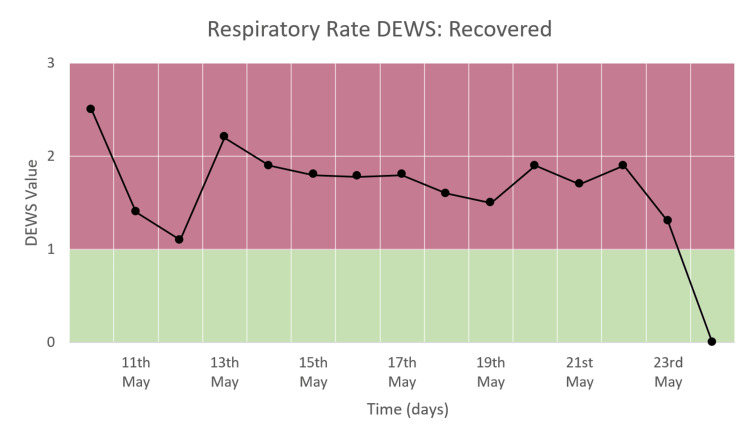
Respiratory rate DEWS of a patient who recovered and was discharged.

The respiratory rate DEWS shows a significant decrease from the time of admission to the time of discharge from the hospital, as seen in Figure 6. The DEWS declines from the range of 2-3 to the range of 1-2 and remains the same for most of the duration of the hospital stay. The score comes down to zero at the time of discharge, corresponding to the normal range.

SpO_2_ DEWS

**Figure 7:**
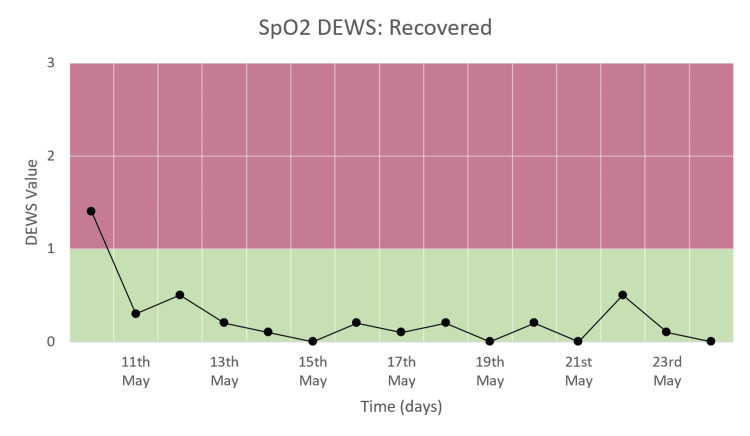
SpO2 DEWS of a patient who recovered and was discharged.

Figure 7 shows that there is a significant decrease in SpO_2_ DEWS in a patient who recovered. The score remained low, in the range of 0-1, until the time of discharge.

Total DEWS

**Figure 8:**
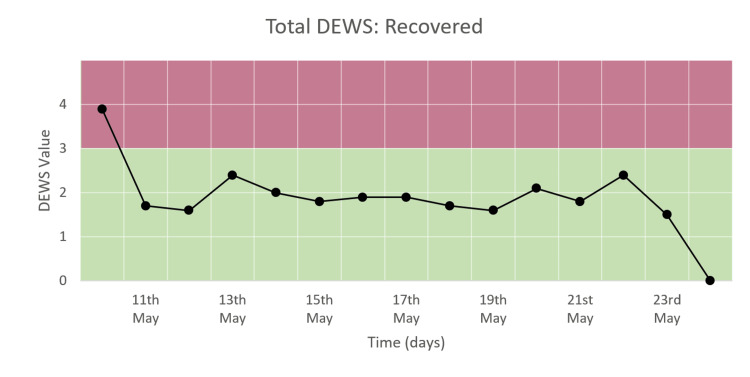
Total DEWS of a patient who recovered and was discharged.

Figure 8 shows a decline followed by consistently low total DEWS over the course of recovery in the hospital. Total DEWS decreased from four to the normal range of 1-3 and came to zero at the time of discharge.

Heart Rate Trend

**Figure 9:**
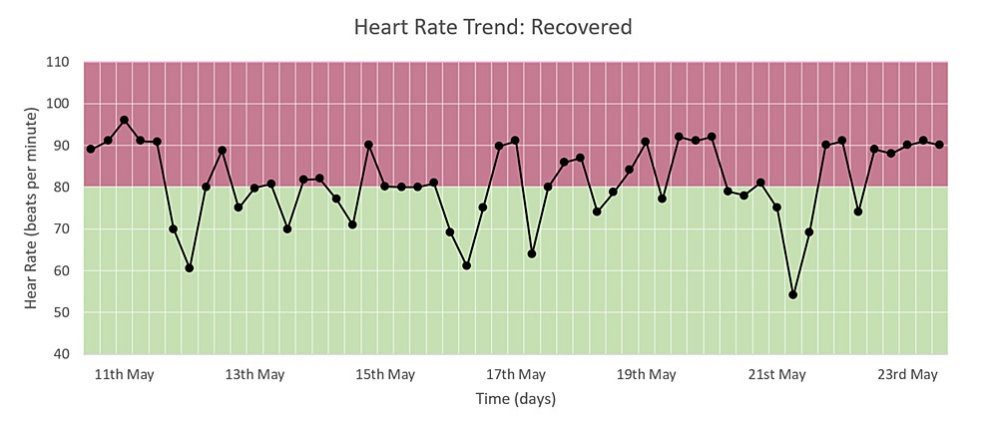
Heart rate trend of a patient who recovered and was discharged.

The heart rate varied from around 50 to 100 bpm during the hospital stay, as seen in Figure 9. No congruous trend is observed, which is consistent with the heart rate DEWS, which showed no change in trend with a constant value of zero (Figure 6).

Respiratory Rate Trend

**Figure 10:**
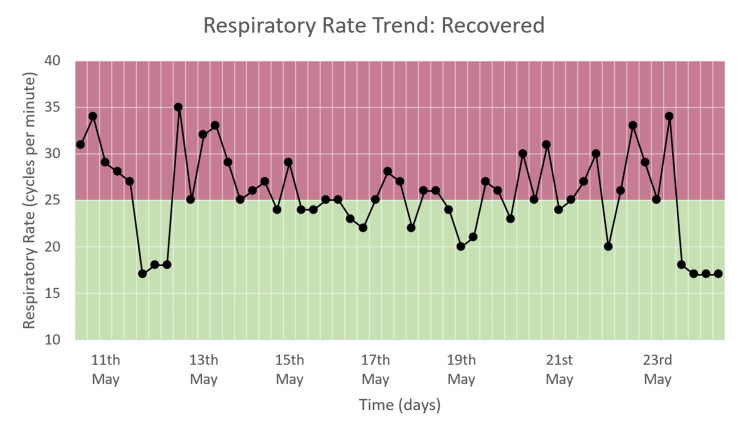
Respiratory rate trend of a patient who recovered and was discharged.

The respiratory rate varied from around 15 to 35 cpm during the hospital stay, as seen in Figure 10. For most of the duration of the hospital stay, RR is between 20 and 30 and comes down to between 15 and 20 by the time of discharge. This trend corresponds to the RR DEWS seen in Figure 6.

DEWS graphs: deteriorated

The following graphs show the various DEWS and vitals trends in a patient who deteriorated and died.

Heart Rate DEWS

**Figure 11:**
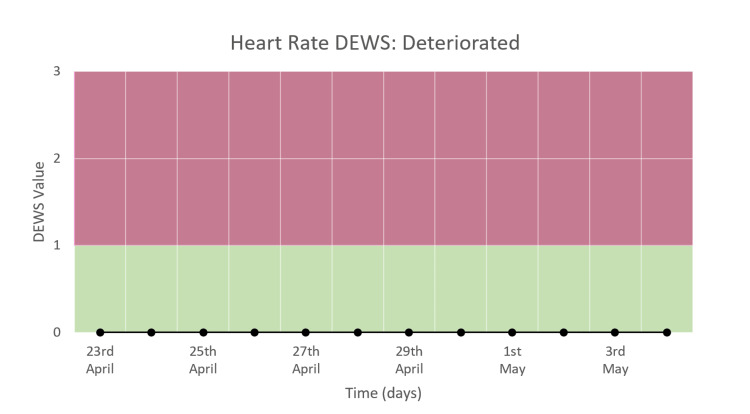
Heart rate DEWS in a patient who deteriorated and died.

Figure 11 shows the heart rate DEWS in a patient who deteriorated. There was no change in the trend during the study period. The DEWS score is consistently zero.

Respiratory Rate DEWS

**Figure 12:**
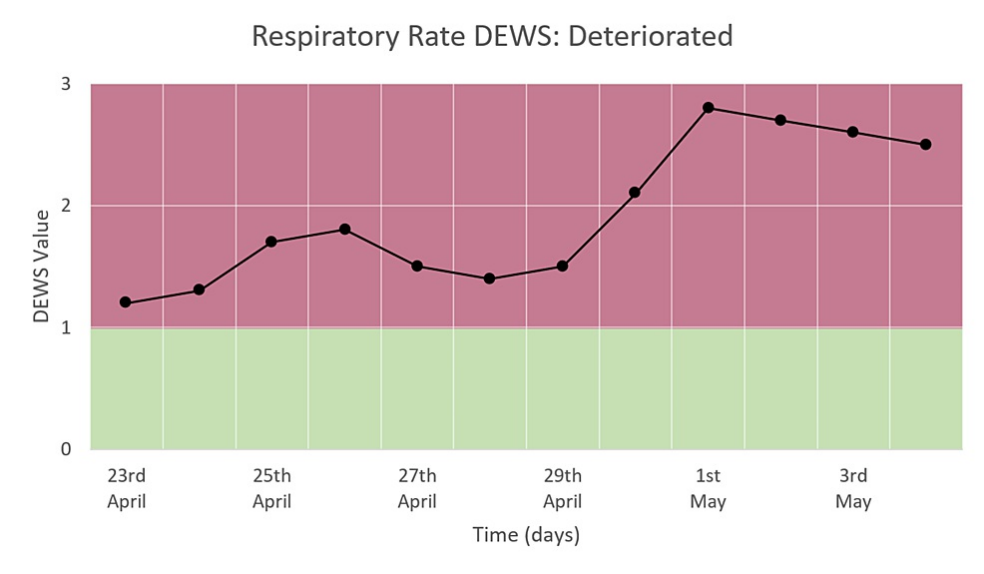
Respiratory rate DEWS in a patient who deteriorated and died.

Respiratory rate DEWS shows a significant increase after admission to the hospital, as seen in Figure 12. The DEWS increases from the range of 1-2 to the range of 2-3 and remains consistently high during the study period.

SpO_2_ DEWS

**Figure 13:**
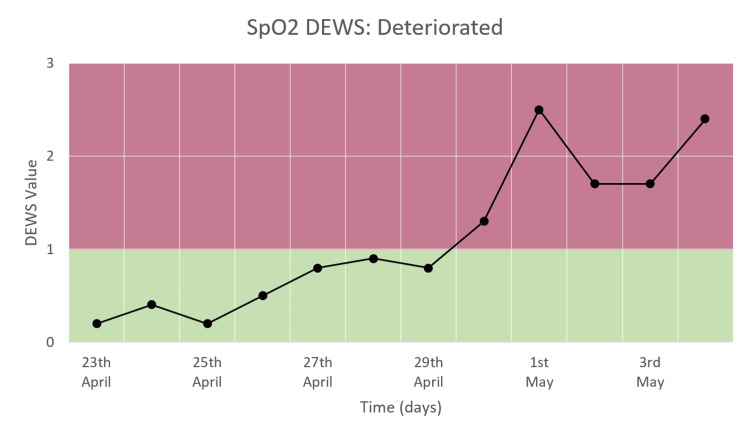
SpO2 DEWS in a patient who deteriorated and died.

Figure 13 shows that there is a significant increase in SpO_2_ DEWS from the time of admission to the time of death. The score remained within the normal range of 0-1 in the first week, after which it increased to and remained in the range of 1-3.

Total DEWS

**Figure 14:**
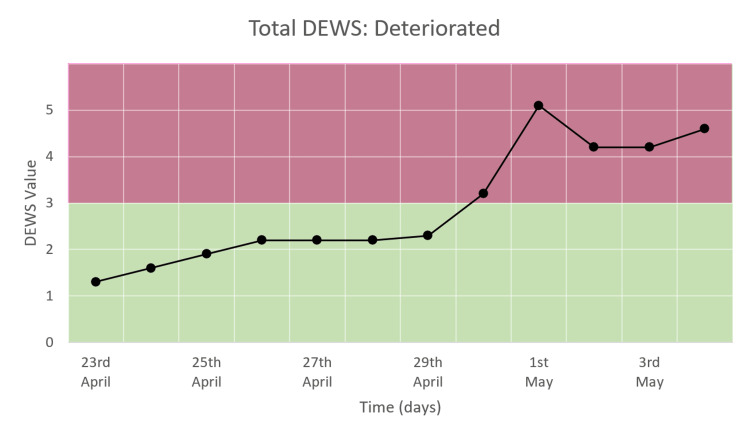
Total DEWS in a patient who deteriorated and died.

Figure 14 shows an incline followed by consistently high total DEWS over the course of the hospital. Total DEWS initially increased within the normal range of 1-3, following which it remained consistently high in the range of 3-5.

Heart Rate Trend

**Figure 15:**
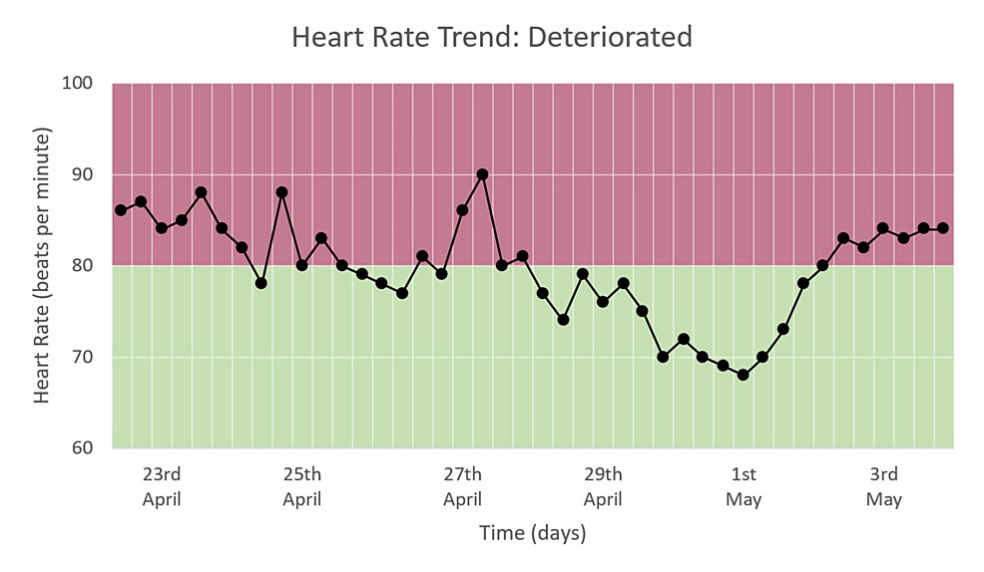
Heart rate trend in a patient who deteriorated and died.

The heart rate varied from around 70 to 90 bpm during the hospital stay, as seen in Figure 15. No congruous trend is observed, which is consistent with the heart rate DEWS, which showed no change in trend with a constant value of zero (Figure 11).

Respiratory Rate Trend

**Figure 16:**
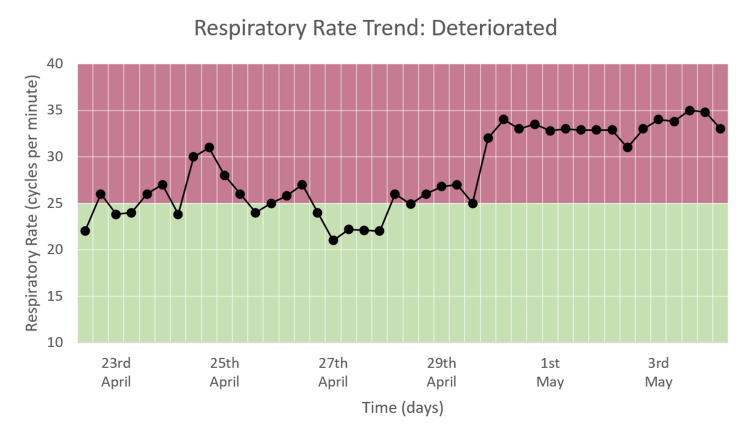
Respiratory rate trend in a patient who deteriorated and died.

The respiratory rate varied from around 20 to 35 cpm during the hospital stay, as seen in Figure 16. During the first week of hospital stay, RR is between 20 and 30, following which it rises to and remains in the range of 30-35 until the time of death. This trend corresponds to the RR DEWS (Figure 12).

SpO_2_ Trend

**Figure 17:**
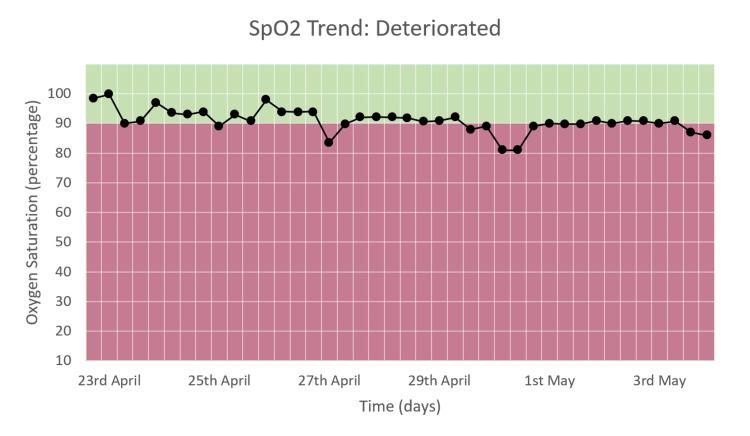
SpO2 trend in a patient who deteriorated and died.

Oxygen saturation percent remained between 90% and 100% during the first five days of the hospital stay, after which SpO_2_ levels slightly declined. From the tenth day of admission, SpO_2_ levels were consistently 90% or below (Figure 17). This is consistent with the SpO_2_ DEWS seen in Figure 13.

Patient outcomes

**Figure 18:**
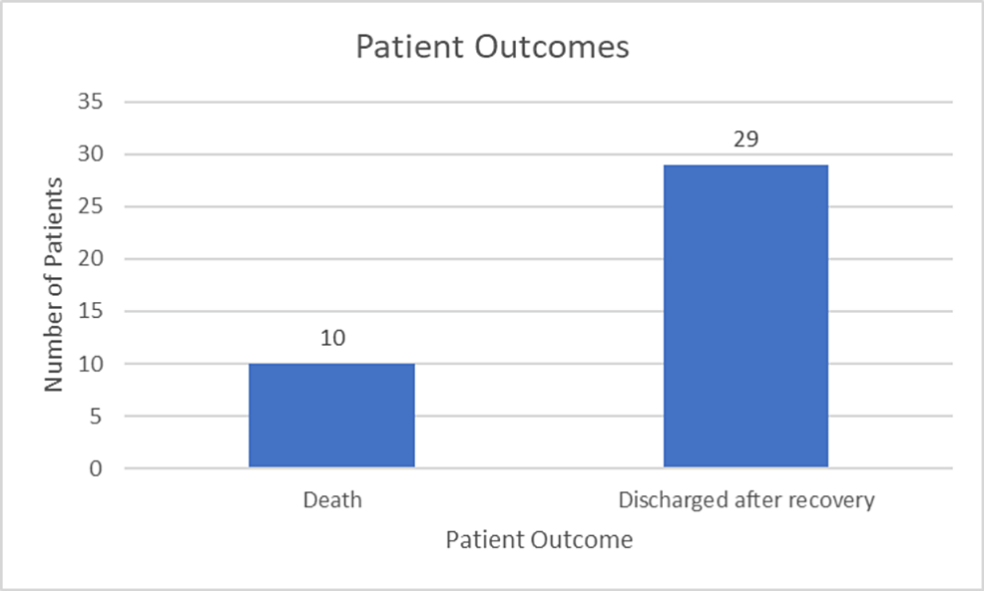
Distribution of patient outcomes.

The outcome of 10 patients was deterioration followed by death, and 29 patients were discharged after recovery, as reported by the healthcare professionals in the ward (Figure 18). Out of the 10 patients who died, five had underlying comorbidities, including hypertension and diabetes mellitus.

## Discussion

We monitored moderate and severe COVID-19 patients and recorded the trends of HR, RR, and SpO_2_ along with their DEWS and total DEWS in patients who recovered from COVID-19 and those who deteriorated and died due to the same. We found that in patients who improved and recovered, the RR trend, RR DEWS, SpO_2_ DEWS, and total DEWS showed significant reductions, while in patients who deteriorated and then died due to the disease, the same parameters showed significant increases followed by consistently high values. The HR trend and HR DEWS remained constant in both outcomes; hence, they did not contribute to the prediction of mortality. These patterns showed that total DEWS was indicative of the outcome of a patient: increased scores were proportional to the risk of mortality and deterioration, while decreased scores were proportional to improvement, indicating recovery. RR DEWS and SpO_2_ DEWS also individually depicted the patient's outcome.

These vital parameters (HR, RR, and SpO_2_) are each scored from 0 to 3, and all the scores are added to make a total score that ranges from 0 to 15. A score of more than 4 requires urgent clinical attention [13]. Dozee is a device developed by Turtle Shell Technologies Private Limited that employs the principle of BCG to monitor these vital parameters. The Dozee sensor sheet records micro-vibrations associated with each cardiac cycle; coupled with additional accessories, it provides an entire overview of patient vitals. Each of these physiological parameters is scored to create a cumulative DEWS. If a patient’s condition crosses set threshold levels for vitals or DEWS increases to a high-risk level, the DEWS System triggers alerts, notifying nurses for urgent clinical review and action [15].

The principle of BCG was overlooked with the development of electrocardiography (ECG) and magnetic resonance imaging (MRI) in the early and late 1900s, respectively. The technique was incorporated into inconvenient and bulky devices that were difficult to use. With recent advancements in technology, BCG has been incorporated into wearable systems that are used for remote monitoring of patients. Although BCG has been shown to have high inter-subject variability, studies show that there are minimal variations in a single patient’s serial readings of BCG signals. Thus, with Dozee, patients serve as their own controls and can be monitored over long periods of time [7].

The development of newer technologies has endowed the medical field with diverse devices, and when coupled with human intellect and judgment, they can help provide an enhanced quality of healthcare. A subset of these devices is of particular interest to us: wearable (remote) sensors. These sensors can perceive a patient’s vital signs in real-time, all the while relaying this data continuously. This allows for frequent monitoring of patients without overburdening healthcare workers. Remote devices can be used as surrogates for monitoring patients, in contrast to the existing in-person model, which requires physicians to physically review vitals bedside [18]. In addition to sensing vital parameters like HR, blood pressure (BP), RR, body temperature, and SpO_2_, these wearable devices are equipped to sense even electrocardiograms (ECG), electromyograms (EMG), and electrodermal activity (EDA). Studies show that continuous monitoring using wearable sensors is well accepted by patients, their attendees, and their healthcare providers [19].

Continuous monitoring was initially limited to operating rooms (ORs), intensive care units (ICUs), and high-dependency units (HDUs). It provides a comprehensive report of variations in vital signs over time, which aids healthcare workers in making appropriate clinical decisions, administering prompt interventions, and deciding when to schedule follow-up visits [15]. Studies show that it effectively estimates vital parameters even in less intensive settings [20].

In conjunction with these remote sensors for continuous monitoring, several EWSs were developed to trigger an alert in order to instigate a rapid response to attend to critically ill patients whose deranged vitals were suggestive of deterioration with a risk of death [21]. Each vital parameter has a score based on its deviation from normal limits, and some EWS combine multiple routinely collected parameters to give a cumulative score, which is an indicator for the in-patient setup [22]. Sensors that combine multiple parameters help bypass traditional manual measurements, thus saving time and offering more dependable next-step measures [10]. These include the Modified Early Warning Score (MEWS), the National Early Warning Score (NEWS), and the Hamilton Early Warning Score. Each has distinct cut-off values for vitals and consciousness levels that set off alarms [21].

Before MEWS, in the late 1900s, some scores were commonly used in ICUs: the Acute Physiology and Chronic Health Evaluation (APACHE) II Score, the Mortality Prediction Model (MDM), and the Simplified Acute Physiology Score (SAPS). These were difficult to calculate and unsuitable for bedside use. MEWS helps in triaging patients, directing necessary interventions toward those who actually need them, and deciding which patients to admit to what ward [10]. Although EWS was developed to detect depreciating patient outcomes, there proved to be a major flaw. The existing EWS did not account for progression with time. Like all things in nature, a patient’s condition is seldom static, which explains the need for a continuous monitoring system that takes into consideration dynamic physiological states. A study by Pimentel et al. reviewed the utility of the hospital-wide alerting via electronic noticeboard (HAVEN) system, which accounted for variability with time, and found that it performed better than previous EWSs [23].

During the COVID-19 pandemic, hospitals began screening in earnest, and a triage system was developed. This was a step in the right direction to identify cases and begin treatment at the earliest possible time. However, the traditional screening method had a major drawback: it exponentially increased the burden on the healthcare system, which manifested in three ways. First, difficulties arose in the timely administration of services due to a rapid rate of increase in the inpatient load; second, the costs incurred during the scheduling of visits and unscheduled in-person screening also went up; third, it condensed large numbers of people in hospitals, clinics, and camps, causing contact with infected individuals and ultimately contributing to the spread of the disease [12,24].

The National Early Warning Score 2 (NEWS2), introduced in 2017, was implemented in the UK during the COVID-19 pandemic. A study by Kostakis et al. showed that NEWS2 was useful to evaluate patient outcomes after a positive RT-PCR test report for SARS-CoV-2 [25]. Gonem et al. confirmed that the use of electronic systems for continuous monitoring permits the comparison of vital parameters at different time points, concluding that dynamic Early Warning Scores perform better at predicting worsening patient outcomes and death. However, this was a retrospective study in a single ward, so it could not comment on the utility in routine clinical practice [26]. These observations are congruent with the DEWS findings obtained by this prospective study, confirming DEWS’ utility in clinical practice. Additionally, the calculation of DEWS is more convenient with contactless monitoring, and the reports are accessible remotely.

The focus of further research should be on DEWS's ability to connect patients to their healthcare providers even outside the hospital setting, providing the advantages of convenience, familial support, and monitoring without hindering their activities of daily living [15]. It would offer a holistic approach to the management of disease, ensuring: convenience - patients can be nursed to health from the comfort of their homes; safety - for patients and healthcare workers alike, by preventing unnecessary exposure; and prevention of death - serving as a predictor of improving or deteriorating conditions, indicating when patients should come to the hospital, prompting early intervention and treatment [27].

There are several limitations to our study. Our study involved a small sample size due to limited device availability. The implications of this single-center study with a small sample size warrant a multi-center study using the same device with a larger sample size. We have not studied the specific impacts of co-morbidities on the clinical deterioration of COVID-19 patients. A drawback of DEWS is that a single score does not suffice when it comes to detecting the deterioration of patients. A study by Blackwell et al. depicted that there are a myriad of reasons that contribute to the outcome of a patient in the ICU, which cannot all be garnered by a single EWS [28]. This warrants the need for a culmination of multiple models to be able to accurately anticipate clinical deterioration in other diseases. Another consideration is that, however advanced the technology is, clinical acumen should also be considered in conjunction with EWS, especially in cases where the EWS does not trigger an alert but the physician or nurse strongly suspects a risk of deterioration [11]. The future trend of DEWS should look into its utility in other chronic diseases that mandate continuous monitoring or where sudden deterioration is expected. Turning the tide against the current rise in various chronic diseases necessitates real-time monitoring of an individual’s physiological parameters.

## Conclusions

We concluded that continuous vitals monitoring of patients and the resulting total DEWS were indicators of improving or deteriorating conditions in patients. The patients who recovered showed a decrease in DEWS, especially respiratory rate DEWS, before they were discharged from the hospital. However, the patients who deteriorated showed a steady increase or consistently high values of total DEWS and respiratory rate DEWS until the time of death.

Continuous monitoring using Dozee in conjunction with DEWS ensures early detection of deterioration, allowing for timely interventions that reduce mortality. It expedites the monitoring process without increasing the burden on the healthcare system. Remote monitoring has emerged as an imperative part of telemedicine in the background of the pandemic, which inculcates real-time vitals data collection from patients that can be accessed by physicians even remotely.
